# The Evolution of Poxvirus Vaccines

**DOI:** 10.3390/v7041726

**Published:** 2015-04-07

**Authors:** Lucas Sánchez-Sampedro, Beatriz Perdiguero, Ernesto Mejías-Pérez, Juan García-Arriaza, Mauro Di Pilato, Mariano Esteban

**Affiliations:** Department of Molecular and Cellular Biology, Centro Nacional de Biotecnología, Consejo Superior de Investigaciones Científicas (CSIC), Madrid-28049, Spain; E-Mails: lsanchez@cnb.csic.es (L.S.-S.); perdigue@cnb.csic.es (B.P.); emejias@cnb.csic.es (E.M.-P.); jfgarcia@cnb.csic.es (J.G.-A.); mauro.dipilato@cnb.csic.es (M.D.P.)

**Keywords:** poxvirus, evolution, vaccines

## Abstract

After Edward Jenner established human vaccination over 200 years ago, attenuated poxviruses became key players to contain the deadliest virus of its own family: Variola virus (VARV), the causative agent of smallpox. Cowpox virus (CPXV) and horsepox virus (HSPV) were extensively used to this end, passaged in cattle and humans until the appearance of vaccinia virus (VACV), which was used in the final campaigns aimed to eradicate the disease, an endeavor that was accomplished by the World Health Organization (WHO) in 1980. Ever since, naturally evolved strains used for vaccination were introduced into research laboratories where VACV and other poxviruses with improved safety profiles were generated. Recombinant DNA technology along with the DNA genome features of this virus family allowed the generation of vaccines against heterologous diseases, and the specific insertion and deletion of poxvirus genes generated an even broader spectrum of modified viruses with new properties that increase their immunogenicity and safety profile as vaccine vectors. In this review, we highlight the evolution of poxvirus vaccines, from first generation to the current status, pointing out how different vaccines have emerged and approaches that are being followed up in the development of more rational vaccines against a wide range of diseases.

## 1. Introduction

The most deadly poxvirus, VARV, was the agent that caused smallpox, a fatal disease for which records go back more than 3500 years [1] and which is suspected to have emerged in 10,000 BCE [2]. Since then, the virus has spread from person to person and from country to country, causing the most severe epidemics in human history, with a death rate of about 30% of those infected. More than 30 years have elapsed since WHO declared smallpox eradicated, but this achievement would have been impossible without the discovery of vaccination and the evolution of poxviruses as vaccines.

The first strategy aiming to fight the smallpox disease was the use of VARV itself as the immunization agent. Variolation, an oriental practice that consisted of inoculating small amounts of material from an infected person to a healthy individual to prevent a natural infection, was used for centuries in the Orient and introduced in Europe by the physician Emmanuel Timoni [3], who described the technique in 1714; the practice was later introduced by Lady Mary Wortley Montagu in the United Kingdom in 1721 [4].

In 1798 the English physician Edward Jenner established a much safer practice, demonstrating that another poxvirus, CPXV—which infected cattle—could be used to prevent smallpox infections in humans. This procedure became known as vaccination, derived from “vacca,” the Latin word for cow. In 1881 Louis Pasteur proposed that vaccination should be the generic term used for preventive inoculations against any animal or human diseases [5].

From 1803 to 1806 the Jennerian vaccination practices and viral strains were expanded to the New World in the Royal Philanthropic Expedition of the Vaccine carried out by the Spanish surgeon Francisco Xavier Balmis, in a programmed expedition under the auspices of King Carlos IV of Spain. In this first expedition in 1803 in the ship *María Pita*, the vaccine was transported to America through arm-to-arm propagation in 22 orphan children. At La Guayra (now Venezuela) the expedition was divided into two groups, one under Salvany, who extended vaccination to South America, and the other led by Balmis, who continued to Cuba and Mexico. From Acapulco the vaccine traveled to Manila (in the Philippines) and then from Macau into China [6,7]. The Spanish expedition was the first-ever mass vaccination campaign, 150 years before the WHO was established. The expedition was successful, and Jenner wrote: “I don’t imagine the annals of history furnish an example of philanthropy so noble, so extensive as this.”

Over time, CPXV and also HSPV were passed through cattle, rabbits, horses and humans and used for vaccinating against smallpox all over the globe. However, at a certain evolutionary point, those viruses were superseded by VACV, another poxvirus whose origin remains unknown, but which eventually became the most studied poxvirus and has been used extensively as a research tool.

The latter half of the 19th century saw the emergence of microbiology and immunology as scientific disciplines. Many of the pioneers in these new sciences used VACV for their studies and vaccine production was introduced into laboratories and taken over by scientists rather than local physicians. This entailed an improvement in the quality of the vaccines, the methods for the distribution and the public health infrastructure, which led to the elimination of endemic smallpox from the industrialized countries of Europe and North America by the early 1950s [8]. Modifications to traditional production and international quality control of vaccines were introduced shortly after the Intensified Smallpox Eradication Program in 1967. Thanks to this program, the last natural case of smallpox occurred in Somalia in 1977, and in 1980 the WHO declared the disease eradicated [9]. To date, smallpox is the only human infectious disease that has been successfully eradicated.

In the early 1980s, recombinant DNA technology revolutionized molecular biology, allowing the insertion of foreign DNA into poxvirus genomes. Early studies by Woodroffe and Fenner indicated in 1960 that homologous recombination could occur between the genomes of two replicating poxviruses [10]. Twenty-two years later, marker rescue studies demonstrated that fragments of genomic [11] and cloned [12] DNA could recombine with the genome of VACV in infected cells. Furthermore, poxvirus expression vectors were described simultaneously in 1982 by the laboratories of Enzo Paoletti [13] and Bernard Moss [14], and recombinant DNA technology quickly became widely used for vaccine development as well as for research in numerous other fields. Thus, the ability to insert heterologous genes into poxvirus genomes deeply improved their vaccination capabilities. Poxviruses were no longer used only as successful smallpox vaccines, but also as vaccines against a wide range of heterologous diseases, namely the hepatitis B surface antigen [15], the hemagglutinin of the influenza virus [16,17], the glycoprotein D of herpes virus [18] and the rabies virus glycoprotein [19], the first foreign antigens and heterologous diseases explored. It is of importance that, as all chordopoxviruses have a similar arrangement of genes, interchangeable promoters and conserved RNA polymerase and transcription factors, the principles developed for VACV expression vectors could be applicable to other poxviruses [20].

In 1990, the genome sequencing of Copenhagen, one of the most studied strains of vaccinia, was published by Paoletti and co-workers [21]. With this knowledge and the ability to insert and delete selective genes, poxviruses have been modified *à la carte* in order to improve their safety and immunogenicity or even their ability to selectively kill tumor cells.

In this article, we review how different poxviruses have evolved in nature and in controlled laboratory environments to generate a wide variety of strains that are being used as vaccine candidates against homologous diseases such as smallpox, heterologous diseases such as rabies, HIV/AIDS, hepatitis C, tuberculosis, malaria and leishmaniasis, among others, or against other complex diseases like cancer. We describe the sequences by which different poxvirus-based vaccines evolved with time, and how genetic manipulation of the poxvirus genome led to the development of vaccine candidates with wide application against human and animal diseases.

## 2. Origin of Vaccination: Cowpox/Horsepox Controversy upon Original Vaccinia Strain

In order to trace, step-by-step, the evolution of poxviruses as vaccine vectors, one of the starting points should be the identification of the original virus used by Jenner and colleagues at early stages of vaccination. In 1796 Jenner vaccinated an eight-year-old boy, James Phipps, with a cowpox lesion from the milkmaid Sarah Nelmes and proved it was effective after challenge against smallpox [22]. From that experiment, the practice of arm-to-arm vaccinations in humans expanded around the world, using cattle to amplify the viral stocks. However, vaccination with the feasible original CPXV was displaced, eventually, with VACV, whose natural host and origin has not been identified yet.

Several hypotheses arose in the past about the origin of VACV and its derivation from the original “*variolae vaccinae*.” It had been proposed that VACV could have derived from VARV, from CPXV, or could be a hybrid of VARV and CPXV viruses that had been genetically selected after the use of contaminated vaccine [23].

VARV could have been altered and transformed into VACV after several years of vaccination from human to human or during passages in animals, but VARV host range genes and studies in animals indicate that this hypothesis should be dismissed [24,25]. VARV has a restricted human replication phenotype that contrasts with the wide host range of VACV [26]. On the other hand, CPXV probably exhibits the broadest host range and the greatest genetic diversity among these poxviruses. Nonetheless, originally the differences between CPXV and VACV were considered too great to make that origin probable [27], and more recent DNA sequencing data follow the same thread of thought [28].

Several factors exacerbated the difficulty of identifying the origin of VACV. The old smallpox vaccines were rarely subjected to clonal purification, and those methods of propagation produced mixtures of viruses called quasispecies [29]. In fact, deep genome sequencing has suggested that modern vaccines are comprised of a complex mixture of different vaccinia viruses [30,31]. Furthermore, the practice of co-cultivating smallpox vaccines with other viruses, including VARV, could have produced recombinant viruses and obscured the origin of VACV strains [8]. In this context, there are few evidences of horizontal gene transfer between orthopoxviruses. One example is the presence of two CPXV-like genes in the Lister VACV strain [32], and another one is represented by a small region of sequence-encoding HSPV-like single nucleotide polymorphisms (SNPs) in the DPP17 Dryvax subclone [29].

Among orthopoxviruses, CPXV isolates—which can be split into five different monophyletic lineages—have the largest genomes, averaging 220 kbp, around 30 kbp larger than VACV; only the genome of HSPV is larger than 200 kbp [33]. These five subtypes of CPXV encode all of the genes present in all other orthopoxviruses, leading to the suggestion that the modern orthopoxviruses have evolved from CPXV through reductive evolution [34].

Historic literature states that the original vaccine strains were derived from CPXV samples; however, Edward Jenner also believed that his vaccine was originally the agent that caused an infection in the heels of horses that he called “grease” and that was suitable for human use after passaging through cows [22,23]. It has also been argued that it could be possible that Jenner confused “grease” (*dermatitis verrucosa*) with horsepox (*variola equina*). In fact, equination was used side by side with vaccination at the early stages of smallpox epidemics. There are several examples of early vaccination with HSPV, for example by Dr. De Carro in Vienna or Dr. Sacco in Milan, who communicated the practice to Jenner [35,36], or by Dr. Loy, who also showed that horses were susceptible to a poxvirus that conferred immunity. Both CPXV and HSPV caused the human immune system to react as if it has been exposed to the smallpox virus, creating long-term immunity because all orthopoxviruses are antigenically related and immunization with nearly any orthopoxvirus can protect against challenge with another orthopoxvirus [37].

Phylogenetic analysis of the conserved regions of poxvirus genomes indicated that HSPV is closely related to sequenced isolates of VACV and rabbitpox virus [38]. Furthermore, it is of interest that experimental infection of horses with VACV produces clinical signs of HSPV [39].

In the most recent article studying poxvirus evolution, the authors claim that the most probable route by which VACV strains might have evolved is from a stock of virus containing an ancestral HSPV-like virus. This conclusion arose after identifying a gene, DVX_213, which seems to have been subject to widespread negative selection in VACV strains [28]. Nonetheless, in order to better understand the relationship between HSPV and VACV it is important to obtain more genomic sequencesofHSPV, an endeavor that is extremely challenging because HSPV seems to be extinct [5].

## 3. First-Generation VACV Vaccines and the Global Smallpox Eradication Campaign

Vaccination is a procedure that has been performed for over 200 years in different countries around the world and without international standardization. In fact, reliable assay methods for quality control were not developed until the 1950s and not implemented until the 1960s. As a consequence, the VACV strains used in different countries differed in their biological properties [8]. These first VACV strains received their names regarding the health agency or the country or region of origin and the most widelyused are summarized in Table 1. In addition, a large number of manufacturers—71 distributed around the world—were involved in the global effort to eradicate smallpox.

**Table 1:** List of the different VACV strains used in the global smallpox eradication campaign.

VACV Strain	Country or Region of Application
New York City Board of Health (NYCBH)	USA
Lister	UK, Europe, Asia, Africa, USA
Temple of Heaven (Tian Tan)	China
Ecuador-Moscow 1963 (EM-63)	Union of Soviet Socialist Republics (USSR), India
Tashkent	USSR
B-15	USSR
Bern	Germany, Austria
Paris	France, Paris, Syria, Turkey
Copenhagen	Denmark
Dairen	Japan
Ikeda	Japan

In the United States all the vaccines against smallpox were derived from a stock supplied to the New York City Health Department in 1856, NYCBH. Using this strain, Dr. Rivers developed two attenuated strains of the virus, CVI-78 and CVII, by passing NYCBH through rabbit testes and chick embryos [40]. These caused less reactogenicity in comparison with the parental strain, but their ability to mediate protection against smallpox was questioned [41,42]. This strain was also distributed to other laboratories, where it received different names, such as IHD, LED-0, Noguchi or WR. It was passaged in different organs such as the brain or testes of rabbits or mice, thus modifying its biological properties. Several studies showed that strains such as WR or IHD presented increased levels of pathogenicity [43] and adverse effects in humans, limiting their use as vaccines of choice in the early steps of the fight against smallpox [8].

Another vaccine was derived from NYCBH after 22 to 28 passages in cows in Wyeth Laboratories (Marietta, PA, USA). This vaccine was called Dryvax^®^ and is a non-clonal vaccine that was prepared in calf lymph and distributed as a freeze-dried vaccine. The nature of non-clonal origin of Dryvax^®^ has been recently characterized. Genetic analysis shows that Dryvax^®^ is a complex of different VACV sub-strains that has been classified in four subgroups according to genome structure analysis [29]. This vaccine is still one of the current USA-licensed smallpox vaccines.

A second vaccine derived from NYCBH and licensed in the USA is Aventis Pasteur Smallpox Vaccine (APSV). APSV was manufactured from 1956 to 1957 and was maintained as a frozen preparation, but clinical studies with this vaccine were stopped when myocarditis cases arose in the vaccination trials [44].

NYCBH was also believed to be the parental strain of EM-63 vaccine, a strain derived from Ecuador that was used in the former USSR and was also widely used in the eradication of smallpox in India [45,46].

The Lister strain, prepared on the skin of sheep, was widely used for vaccination against smallpox from 1892 because it produced pocks on the chorioallantoic membrane that were easier to count in comparison with the other VACV strains, and because the WHO International Reference Center later produced seed lots of this strain for distribution to vaccine producers in developing countries [8]. The commercial Lancy-Vaxina (Berna Biotech) is derived from this strain, and the formulation of the vaccine is a lyophilized product prepared from calf lymph [47].

Tian Tan virus was the most extensively VACV strain used to vaccinate against smallpox in China since 1926. The name of “Tian Tan” was acquired because the virus was isolated in Beijing’s Temple of Heaven, where the Central National Epidemic Bureau was initially housed. The virus was used in China from 1926 to 1954 and from 1960 to 1980, being the Russian strain of vaccinia used between 1955 and 1960 [48]. The legend tells that the vaccine was isolated by Mr. Qi Changqing from a patient with smallpox and then passaged in the skin of monkeys, rabbits and cows. However, this story lacks scientific credibility because VARV infection is restricted to humans and does not contain the host range genes needed to infect monkeys, rabbits or cows. Moreover, recent genomic sequencing demonstrates that Tian Tan is clearly a VACV that shares a common origin with Copenhagen strain, and it is different from monkeypox (MPXV), VARV or HSPV [48].

The intensified Eradication Campaign against smallpox started in 1967 and no particular strain was officially recommended but, in response to inquiries, the Smallpox Eradication Unit advised that either the Lister or the NYCBH strains should be used. Additionally, the potency and safety of the different vaccines were standardized; lyophilization was recommended and vaccine batches might contain at least 1 × 10^8^ pock-forming units per mL [8]. Other VACV strains used in early vaccination campaigns against smallpox were Ankara (used in Turkey), Aosta (Italy), Bohemia (Czechoslovakia), Bordeaux (Africa and Portugal), Massachusetts 999 (Argentina), Gam (USSR), MRIVP (USSR), Per (USSR), Williamsport (USA), LMC (UK), Hamburg (Germany), Sweden (Sweden), Finland (Finland), Patwadanger (India), Vienna (Bulgaria), Spain (Spain), Tom (USSR) and Chambon (France and Africa) [8,49].

The last natural infection of smallpox occurred in Somalia in 1977. Eradication was possible due to vaccination, but also because to date no animal reservoir for smallpox other than humans exists [50]. Since the eradication, VARV is officially retained at two WHO collaborative centers: the Centers for Disease Control and Prevention (CDC), in Atlanta (Georgia, USA), and the State Research Center of Virology and Biotechnology (VECTOR), in Novosibirsk (Russian Federation).

Although smallpox has been eradicated as a public health threat it could potentially reemerge as a bioterrorist threat. The risk scenario includes other animal poxviruses and genetically engineered manipulations of poxviruses. Thus, for preventing this potential risk and due to the side effects of the first-generation VACV vaccines, safer VACV strains had to be improved and developed for the post-eradication era.

## 4. Second-Generation VACV Vaccines

In order to standardize procedures, control possible microbial contamination and avoid sensibilization to the allergenic animal proteins that accompanied the vaccine, the use of live animals for the growth of the different vaccines was substituted by tissue culture systems or embryonated chicken eggs. These second-generation vaccines are listed in Table 2.

Lister was the first VACV strain used for the production of cell-cultured derived smallpox vaccines, being passaged in rabbit kidney cells, in the chorioallantoic membrane of chicken embryos (CE) or in primary cells derived from chicken embryos.

Thus, the first second-generation Lister-based vaccine, RIVM, was produced in 1960 using rabbit kidney cells [51]. The virus was passaged directly from calf lymph vaccine to cells, and no further passages were performed for the generation of this vaccine. Freeze-dried vaccine demonstrated similar take rates and neutralizing antibodies to the calf lymph-derived vaccine [8]. This vaccine has been used in clinical trials in Netherlands and Indonesia without producing severe complications [52].

**Table 2:** List of the different second-generation VACV-based vaccines.

Strain	Vaccine	Cell Culture	References
Lister	RIVM	Rabbit kidney cells	[51]
	Israel	Chorioallantoic membrane of CE	[53,54]
	Lister/CEP	CE cells	[55]
	Elstree-BN	CE cells	[56,57]
NYCBH	CCSV	MRC-5 cells	[58]
	ACAM1000	MRC-5 cells	[59]
	ACAM2000	VERO cells	[60]
	CJ-50300	MRC-5 cells	[61]
	WR	Rabbits, mice, cell cultures	[43]

Abbreviations: RIVM: Rabbit Lister Vaccine; CEP: Chicken embryo primary cells; CE: Chicken embryos; BN: Bavarian Nordic; CCSV: Cell Culture Smallpox Vaccine; WR: Western Reserve.

Lister strain grown in chorioallantoic membranes of CE has been used in the military forces of Israel in the 1990s and 2000s with no severe complications observed [53,54]. Furthermore, Sanofi Pasteur developed another second-generation VACV vaccine, passaging a batch of the first-generation Lister vaccine during three passages in CE primary cells (CEP). This vaccine, Lister/CEP, was similar in immunogenicity and safety in comparison with the parental first-generation Lister vaccine [55]. In addition, Bavarian Nordic (BN) also manufactured a vaccine using Lister strain, called Elstree-BN, that was passaged in CE cells and demonstrated safety and immunogenicity in preclinical studies in monkeys [56] and in human clinical trials conducted in 2004 [57]. This vaccine was also prepared on chicken embryo fibroblast (CEF) cells in Japan before smallpox eradication and showed an adequate safety profile, but the effectiveness was not well documented [45].

Several second-generation VACV vaccines were also prepared using NYCBH as the seed strain. The first candidate was grown in cell cultures in 1968 and was used in clinical trials in the U.S. Army that had to be stopped due to the absence of adequate “take” rates observed [2]. Nonetheless, from that stock, another cell-cultured stock was developed in MRC-5 cells and received the name of Cell-Cultured Smallpox Vaccine (CCSV). In 2002, a head-to-head phase I clinical trial comparison with Dryvax^®^ was performed and dilutions up to 1/50 of CCSV vaccine showed a 100% take rate and no statistical significance differences in immunogenicity in comparison with Dryvax^®^ [58]. Dryvax^®^ was used to vaccinate military personnel in 2002 [62].

Another vaccine candidate derived from NYCBH is ACAM2000 (from Acambis), a vaccine derived from a clone isolated from Dryvax^®^. Originally six clones were isolated and their safety was evaluated in suckling mice and in rabbits. Significant differences in neurovirulence were observed among the different clones; CL2 is a clone with reduced neurovirulence that still maintains the same lesion size when compared with the Dryvax^®^ vaccine [59]. This clone was selected and grown first in MRC-5 cells (ACAM1000) and, later on, in VERO cells generating the ACAM2000 vaccine [60]. Preclinical studies demonstrated that this strain was less neurovirulent in comparison with Dryvax^®^, but demonstrated similar immunogenicity in phase I clinical trials. Nonetheless, in phase II and III clinical trials, Dryvax^®^ and ACAM2000 caused myocarditis associated with the vaccination [59,60]. The Food and Drug Administration (FDA) approved ACAM2000 in 2007 as a vaccine against smallpox for human use and Sanofi Pasteur manufactured the vaccine.

NYCBH has also been used as a parental seed strain for the development of another second-generation VACV vaccine termed CJ-50300, which was obtained after passages in MRC-5 cells in South Korea. Compared to the first-generation Lancy-Vaxina vaccine, it showed similar reactogenicity, immunogenicity and neurovirulence in preclinical trials [47]. Moreover, a phase I clinical trial showed overall rates of 100% in cutaneous “take” reaction and humoral and cellular immunogenicity in CJ-50300 vaccinees, with no serious adverse reactions being observed. However, one case of possible generalized vaccinia infection occurred in one of the studied groups [61].

Other strains have been also derived from NYCBH such as Western Reserve (WR), a neurovirulent strain that has a wide history of passages; first in rabbits, followed in mice and in cell cultures [43], and Duke (isolated from a vaccinated patient that received Dryvax^®^ vaccine [63]). As new research proves, VACV IHD-J, “International Health Department,” also shares a common ancestor with Dryvax^®^, *i.e.*, NYCBH [28].

All these studies with second-generation vaccines demonstrate that although cell-cultured vaccines improved the control and the standardization that were lacking in previous vaccines, the use of replication-competent strains of VACV represents associated risks and serious adverse events that still have to be controlled.

Several first- and second-generation poxvirus strains expressing different heterologous antigens have been used as vaccine candidates against a wide range of diseases. Table 3 summarizes the most relevant recombinant poxviruses used for these purposes.

Other members of the poxvirus family have also been extensively used as vaccine vectors against homologous and heterologous diseases (see Table 4 and Table 5). There are several examples of vaccines based on avipoxvirus, suipoxvirus, capripoxvirus, leporipoxvirus and parapoxvirus, which belong to the *Chordopoxvirinae* subfamily.

**Table 3:** Preclinical studies using first- and second-generation poxvirus strains as vaccine candidates against different viral, bacterial and parasitic infectious diseases.

Poxvirus Strain	Target Pathogen or Disease	Heterologous Antigen	Status	References
Lister	Hepatitis B	HBsAg	preclinical	[64,65]
	Cystic echinococcosis	*Echinococcus* *granulosus* EG95	preclinical	[66]
	Lassa Fever	Nucleocapside	preclinical	[67]
Wyeth	Influenza A	HA, NA, M1, M2 and NP from H5N1	preclinical	[68,69]
	Hepatitis B	preS2-S	preclinical	[70]
	Rinderpest	F and HA	preclinical	[71]
	Lassa fever	Glycoprotein	preclinical	[72]
	Anthrax	PA of *Bacillus anthracis*	preclinical	[73]
Copenhagen	Rabies	Glycoprotein	preclinical	[74,75]
	HCMV	gB	preclinical	[76]
	RVHD	Capsid protein (VP60)	preclinical	[77]
	Measles	HA, F, NP	preclinical	[78]
	Equine Herpesvirus	GP13	preclinical	[79]
WR	Malaria	PYCS, Pf155/RESA, GLURP	preclinical	[80,81,82]
	Influenza	HA, NP	preclinical	[16,83]
	HIV/AIDS	ENV, ENV (TAB13)	preclinical	[84,85,86]
	Leishmaniasis	LACK	preclinical	[87,88]
	Hepatitis B	HBsAg	preclinical	[89]
	Rabies	Glycoprotein	preclinical	[74]
	Anthrax	PA	preclinical	[90]
	Japanese Encephalitis Virus	Structural proteins	preclinical	[91]
	Rinderpest	F, HA	preclinical	[92,93]
	Measles	F, HA	preclinical	[94]
	Brucella	18 kDa	preclinical	[95]
	Respiratory Sincitial Virus	F, G	preclinical	[96]
	Feline Infectiuos Peritonitis	Fusogenic Spike Protein	preclinical	[97]

Abbreviations: HBsAg: Hepatitis B Virus Surface Antigen; HA: Hemagglutinin; NA: Neuraminidase; NP: Nucleoprotein; F: Fusion protein; PA: Protective antigen; HCMV: Human Cytomegalovirus; gB: Glycoprotein B; RVHD: Rabbit Viral Hemorrhagic Disease; ENV: Envelope; HBsAg: Hepatitis B Virus Surface Antigen; PYCS: Plasmodium Yoelii Circumsporozoite; GLURP: Glutamate Rich Protein; LACK: Leishmania homolog of activated C Kinase.

Avipoxviruses (APVs) belong to the *Chordopoxvirinae* subfamily of the *Poxviridae* family. They infect and cause diseases in poultry, pets and wild birds, are transmitted via biting insects and aerosols and are usually named on the basis of the bird species from which the virus was first isolated and characterized [98]. APV infections have been reported to affect over 232 species in 23 orders of birds [99]. However, the knowledge of the molecular and biological properties of APVs is largely restricted to canarypox virus (CNPV) and fowlpox virus (FWPV), for which full genome sequences are available [100,101]. Despite the shorter FWPV genome, molecular comparisons show that CNPV and FWPV share 55–71% amino acid identity, significant gene-sequence rearrangements, deletions and insertions [101]. CNPV exhibits a broader tissue tropism in the permissive avian hosts than FWPV, generally associated with higher mortality rates [102]. Both viruses have been described as unable to replicate and disseminate infection in non-human primates and humans [103], but some studies have shown replication of FWPV in non-permissive mammalian cell cultures by the presence of infectious viral particles [104] or the occasionalpresence of immature forms and mature intracellular virus in infected cells [105]. However, a recent study has demonstrated that despite the detection of mature virions in FWPV-infected VERO cells, the new progeny was not infectious [106]. Due to their natural host-range restriction to avian species [103,105,107], their efficient expression of heterologous genes also in human cells [108], and their ability to induce antigen-specific humoral and cellular immune responses [109,110], CNPV and FWPV represent alternative and safer vectors. In this context, several recombinant APVs have been evaluated as vaccine candidates against a wide range of infectious diseases and other APV-based vaccines have been licensed for commercial veterinary use against some animal infections; it is likely that such vaccines will also be used against human diseases in the future [111]. Table 4 summarizes the most relevant recombinant avipoxviruses used as vaccine vectors against different diseases.

**Table 4:** Vaccine applications of avipoxvirus-based vectors.

Pox Strain	Target Pathogen or Disease	Heterologous Antigen	Status	References
**Viral Infections**				
CNPV	HIV/AIDS	HIV-1_SF2_ Env	preclinical	[112]
FWPV	HIV/AIDS	HIV-1_SF2_ Env	preclinical	[112]
		SIV_mac239_ Gag/Pol, SIV_89.6P_ Env, Gag/Pol, Env, Tat/Rev (clade B), Gag/Pol, Env, Tat/Rev (clade A/E), IFN-γ, IL-2	preclinical	[113,114,115,116,117,118]
		HIV-1 TAB9 multiepitopic polypeptide	preclinical	[119]
		MEG(4): multi-epitope gene (4 HIV-1 B cell epitopes), HIV-1 p24, MEG(25): multi-epitope gene (25 HIV-1 CTL epitopes)	preclinical	[120]
		HIV_CN_ gp120, IL-2	preclinical	[121]
		Gag, Env (clade D), cholera toxin B subunit	preclinical	[122]
		HIV-1_SF2_ Gag, Pol, HIV-1_BH10_ Env, IFN-γ	preclinical	[116,123]
		HIV-1_SF2_ Gag, Pol, IFN-γ	clinical	[124]
		Gag and Pol (clade B)	clinical	[125]
		Gag/Pol, Env, Tat/Rev (clade A/E)	clinical	[126]
		Env/Gag, Tat/Rev/Nef-RT (clade B)	clinical	[127]
	NDV	F and HN	licensed for commercial veterinary use (chickens)	[128,129,130,131,132,133,134,135,136]
	ILTV	gB (+AE)	licensed for commercial veterinary use (chickens)	[137,138,139]
	NDV + ILTV	F and HN (NDV) + gB (ILTV)	preclinical	[128]
	IBV	S1, S1 + IFN-γ, S1 + IL-18	preclinical	[140,141,142,143]
	AEV	AE (+LT)	licensed for commercial veterinary use (chickens)	[137]
	AIV	native or synthetic HA, HA and/or NP, HA + IL-18 or IL-6, NA, HA + NA, LPAIV insert	preclinical	[144,145,146,147,148,149,150,151,152,153,154,155,156,157,158,159,160,161]
	IBDV	VP2, VP2-VP4-VP3	preclinical	[162,163,164,165,166]
	MDV	glycoproteins B, E, I, H and UL32, pp38	preclinical	[167,168,169,170,171]
	Rabies virus	Glycoprotein	preclinical	[110,172]
	HPV	L1 structural protein, E6 and E7 oncoproteins	preclinical	[173,174]
	FMDV	capsid and 3C protease, P1, 2A and 3C, IL-18	preclinical	[175,176]
	CSFV	E0	preclinical	[177]
	DHBV	DHBc and Pre-S/S antigens	preclinical	[178]
	PRRSV	GP5/GP3, IL-18	preclinical	[179]
	TRTV	F	preclinical	[180]
	CDV	H and F antigens of RPV	preclinical	[181]
	HEV	native hexon	preclinical	[182]
	MeV	F	preclinical	[183]
	Smallpox	VACV L1, A27, A33 and B5	preclinical	[184,185]
	APV	-	preclinical	[186,187,188]
**Bacterial diseases**				
FWPV	*Mycoplasma gallisepticum*	40 k and mgc gene segments	licensed for commercial veterinary use (chickens)	[189]
**Parasitic diseases**				
FWPV	*Eimeria tenella*	rhomboid gene	preclinical	[190]

Abbreviations: CNPV: Canarypox virus; FWPV: Fowlpox virus; NDV: Newcastle disease virus; ILTV: Infectious laryngotracheitis virus; IBV: Infectious bronchitis virus; AEV: Avian encephalomyelitis virus; AIV: Avian influenza virus; IBDV: Infectious bursal disease virus; MDV: Marek’s disease virus; HPV: Human papilloma virus; FMDV: Foot-and-mouth disease virus; CSFV: Classical swine fever virus; DHBV: Duck hepatitis B virus; PRRSV: Porcine reproductive and respiratory syndrome virus; TRTV: Turkey rhinotracheitisvirus; CDV: Canine distemper virus; RPV: Rinderpest virus; HEV: Hemorrhagic enteritis virus; MeV: Measles virus; F: Fusion protein; HN: Hemagglutinin-neuraminidase proteins; gB: Glycoprotein B; HA: Hemagglutinin; NP: Nucleoprotein; NA: Neuraminidase.

Furthermore, other poxvirus vectors of the *Chordopoxvirinae* subfamily, such as the orthopoxvirus raccoon poxvirus, parapoxvirus, capripoxvirus, suipoxvirus and myxomavirus have been widely used as vaccine candidates against several animal and human diseases (see Table 5), showing good levels of safety and immunogenicity.

**Table 5:** Preclinical studies using other pox vectors as vaccine candidates against different viral, bacterial and parasitic infectious diseases.

Pox Strain	Target Pathogen or Disease	Heterologous Antigen	Status	References
Raccoon poxvirus	Influenza A	HA and NA from H5N1	preclinical	[191]
	Bubonic plague	F1 capsular antigen of *Yersinia pestis*	preclinical	[192,193,194,195,196]
	Rabies	Glycoprotein	preclinical	[19,197,198,199,200]
		Internal structural NP	preclinical	[198,201]
	Feline panleukopenia	VP2	preclinical	[199,202]
	FIPV	Nucleocapsid	preclinical	[203]
Parapoxvirus (orf)	Influenza A	HA or NP from H5N1	preclinical	[204]
	Rabies	Glycoprotein	preclinical	[205]
	PRV	Glycoproteins gC and/or gD	preclinical	[206,207,208]
	Borna disease	NP p40	preclinical	[209]
	RVHD	VP1 (VP60)	preclinical	[210]
	CSFV	E2 glycoprotein	preclinical	[211]
Capripoxvirus	PPRV	F or HA	preclinical	[212,213,214,215,216,217,218]
	HIV/AIDS	HIV-1 subtype C Gag, reverse transcriptase, Tat and Nef	preclinical	[219,220]
	Rift Valley fever	Glycoproteins Gn and Gc	preclinical	[221,222]
	Rinderpest	F or HA	preclinical	[223,224,225,226,227]
	Bluetongue	VP2, VP7, NS1 and NS3	preclinical	[228,229]
	Rabies	Glycoprotein	preclinical	[230]
Suipoxvirus	PCV2-associated disease	IL-18 + Cap, Cap	preclinical	[231,232]
	SEZ	M-like protein (SzP)	preclinical	[232]
		MRP of *S. suis* type 2 (SS2)	preclinical	[233]
	Swine influenza	HA1 from H3N2 and H1N1	preclinical	[234,235]
		HA1 from H3N2	preclinical	[236]
		HA1 from H1N1	preclinical	[236]
Myxomavirus	Bluetongue	VP2	preclinical	[237]
	Feline calicivirus disease	Cap	preclinical	[238,239]
	Influenza	HA from H5N1	preclinical	[240,241]
	RVHD	Capsid protein (VP60)	preclinical	[242,243,244,245]

Abbreviations: HA: Hemagglutinin; NA: Neuraminidase; NP: Nucleoprotein; FIPV: Feline infectious peritonitis virus; F: Fusion protein; PCV2: Porcine circovirus type 2; Cap: Capsid protein; MRP: Muramidase-related protein; PRV: Pseudorabies virus; PPRV: Peste des petits ruminants virus; CSFV: Classical swine fever virus; SEV: *Streptococcus equi* ssp. Zooepidemicus; RVHD: Rabbit Viral Hemorrhagic Disease.

## 5. Third-Generation VACV Vaccines: Evolution through Several Passages in Cultured Cells

Given the unsatisfactory safety profile of VACV second-generation vaccines, attention has shifted to third-generation vaccines, obtained after serial passages in cell culture [37]. Thus, multiple extensive passages of a parental vaccine strain in cultured cells is a useful strategy for attenuating VACV through the generation of random mutations and deletions. Examples of this strategy are different VACV strains that are used as vaccine candidates, such as Lister clone 16m8 (LC16m8), Dairen I strain (DIs), M65 and M101, Modified Vaccinia Virus Ankara (MVA) and several attenuated avipoxviruses.

### 5.1. LC16m8

LC16m8 was obtained in the late 1970s in Japan by passaging the parental Lister strain 36 times in primary rabbit kidney (PRK) epithelial cells at low temperature (30 °C), followed by isolation of one clone (LC16) that grows to the lowest titer in monkey kidney VERO cells; this was passaged six additional times in PRK cells to obtain the clone LC16m0 from the latter stock. Then, this clone was passaged three more times in PRK cells to generate the clone LC16m8 from the final stock [246,247]. LC16m8 replicates poorly in VERO cells, and formed small plaques in chick chorioallantoic membranes (CAM), PRK and RK13 cells. Thus, while LC16m8 can grow and produce infectious particles, it spreads poorly in cell culture. Compared to the original Lister strain, LC16m8 is temperature-restricted and displays limited host range, lower pathogenicity and adverse effects in animal models [247,248].

LC16m8 contains a frame-shifting single nucleotide deletion in the *B5R* gene [30,249], which encodes an extracellular enveloped virus (EEV) protein (B5) essential for EEV formation. Analysis of the LC16m8 full-genome sequence showed that there are no large deletions compared to the parental Lister strain [30].

LC16m8 has been shown to induce protective immunity against orthopoxvirus challenge in mice [30,250,251], rabbits [250] and non-human primates [252,253]. Moreover, LC16m8 is a safe and immunogenic attenuated smallpox vaccine in immunodeficient mice [254] and vaccinia-naive humans [255,256]. However, there are two important main concerns about this vaccine. First, since the key attenuating mutation in *B5R* is a one base deletion that results in a frame-shift and early truncation of the B5 protein, the virus can revert back to wild type during growth [257]—although, to avoid this phenomenon, a new version of LC16m8, with a complete deletion in the *B5R* gene, has been generated [257]. The second concern is related to the fact that VACV B5 protein is the primary target antigen for generating neutralizing antibodies against EEV [258]. Thus, due to the presence of a mutation in *B5R*, LC16m8 failed to induce either EEV-neutralizing antibodies or antibodies to B5 in humans [259], a feature that may make LC16m8 a less efficient vaccine for protection against poxviruses. It remains to be seen whether this strain induces similar levels of neutralizing antibodies against VARV than other vaccine strains such as Dryvax^®^.

Nevertheless, the combination of the deletion in the *B5R* gene (which causes the lack of anti-VACV vector immunity) with the insertion of heterologous antigens in the VACV TK or HA loci is a good strategy for using LC16m8 as a vaccine vector against infectious diseases. Thus, it has been reported that an attenuated recombinant LC16m8 expressing clade B HIV-1 Env [260] or SARS-CoV spike protein [261] was able to induce robust HIV-1-specific humoral and T cell immune responses or SARS-specific neutralizing antibodies in vaccinated mice and rabbits, respectively.

Thus, LC16m8 is one of the safest live, attenuated, replication-competent vaccines; it is the sole smallpox vaccine licensed in Japan and was recently recommended by the WHO as one of the preferred WHO smallpox vaccines to stockpile. Furthermore, it is a promising vaccine vector against infectious diseases.

### 5.2. Dairen I Strain (DIs)

VACV DI strain was generated after 13 successive passages of parental Dairen strain in one-day-old eggs [262]. DIs forms small plaques in CAM, growing well only in chick embryo fibroblast (CEF) cells, but is unable to grow in most mammalian cells. DIs is a highly restrictive host range mutant that contains a great deletion of 15.4 Kb in the left terminal region of the VACV genome, which results in the loss of 19 putative ORFs from genes *C9L* to *K5L*, including host-range genes *K1L* and *C7L* [263].

Insertion of HIV-1 Gag gene in the deleted region of DIs induced high levels of cytotoxic T lymphocytes in immunized mice [263]. Furthermore, a recombinant VACV DIs expressing simian immunodeficiency virus (SIV) Gag and Pol antigens induces SIV-specific cellular and humoral immuneresponsesin mice [264,265] or immunized non-human primates [266,267]. These results suggest that recombinant VACV DIs is a safe, efficient, transient replication-deficient viral vector, which can be used in a vaccine regimen for HIV-1 vaccine development.

### 5.3. M65 and M101 Virus

M65 and M101 strains of VACV were generated in the 1980s after 65 and 101 passages, respectively, of Friend erythroleukemia (FEL) cell line persistently infected with WR strain [268]. During persistent infections of FEL cells, these mutants suffered large deletions of about 8 MDa at the left terminus of the viral genome [269] and alterations in some of the structural proteins with roles in the morphogenetic pathway, exhibit a small plaque size phenotype compared with WR parental virus, are highly attenuated and maintain replication capacity in some mammalian cell lines [270]. Their genomes have been recently sequenced, showing multiple point mutations and specific gene deletions [271]. Recombinants based on these and other mutants at early passages in FEL cells and expressing parasite antigens for malaria and leishmaniasis have been shown to elicit protection after challenge with parasites in prime/boost regimens in mice [268,271,272].

### 5.4. Modified Vaccinia Virus Ankara (MVA)

MVA is a highly attenuated VACV strain generated in Germany in the 1960s by passaging the Turkish smallpox vaccine chorioallantoic VACV Ankara (CVA) strain more than 570 times in primary CEF cells [273,274]. During these extensive serial passages in cell culture, MVA lost nearly 15% (around 30 Kb) of the parental CVA genome (containing several point mutations and large deletions compared to CVA), mainly in both left and right terminal regions with many of the genes deleted involved in the host range and in the modulation of host immune responses [275,276]. As a result of this dramatic evolutionary genomic modification, MVA has lost the ability to produce infectious progeny virus in almost all mammalian cell lines, including human cells [274,276,277,278], replicating efficiently only in CEF and BHK-21 cells. Thus, in most of the cells MVA produces early, intermediate and late proteins, but only immature virions are formed [279]. Because of the inability to replicate in human cells, MVA would likely be safe to administer to people who have conditions that would not allow routine smallpox vaccination. In fact, MVA was used as a safe highly attenuated smallpox vaccine in the last decades of the smallpox eradication campaign (1968–1980), being inoculated into more than 120,000 people in Germany with no adverse side effects [274,280], although its efficacy against smallpox remains untested.

Since then, MVA has been widely studied as a third-generation smallpox vaccine [281], able to induce antibody responses similar to Dryvax^®^ [282] as well as protection in mouse [283] and non-human primate challenge models [56,284,285,286]. However, high doses or multiple doses of MVA have to be administrated to elicit immune protection, compared with other smallpox vaccines such as Dryvax^®^ [283,287,288]. Nevertheless, this protection elicited by MVA is more rapid than the one induced by the fully replication-competent vaccine Dryvax^®^ [286], mainly due to the induction of a more rapid immunity and an activation of the innate immune responses. Furthermore, MVA lacks several VACV immunomodulatory genes involved in evasion of the host immune responses, such as soluble receptors for type I and II IFNs, cytokines and chemokines [277,289], whose absence allows an enhanced antigen presentation and immunogenicity. In fact, deletion of innate immune evasion genes leads to an increase in proinflammatory cytokines and migration of immune cells [290,291,292], which have a great influence on their ability to elicit adaptive immunity.

Thus, MVA has been evaluated as a smallpox vaccine in different animal models and several human clinical trials and was found to be safe and immunogenic without developing clinical disease [2,283,285,287,293,294,295,296,297,298,299,300]. Although the MVA vaccine has not been tested directly in humans for efficacy against VARV, it has being tested against monkeypox triggering protection. In terms of MVA’s registration as a smallpox vaccine, the European Medicines Agency registered the vaccine as Imvanex and Health Canada also registered the vaccine for persons 18 years and older, while in the USA it is under evaluation by the FDA.

Among poxviruses, MVA is the tip of the iceberg, being one of the most promising poxvirus vectors (reviews in [37,301,302,303,304,305,306,307,308,309,310]). Enormous effort has been put into the use of MVA as a vaccine vector, with several preclinical and human clinical trials developed using MVA as a vaccine candidate against an extensive number of infectious diseases, such as HIV/AIDS, malaria, tuberculosis, hepatitis C and cancer, among many others. Table 6 summarizes the use of MVA in preclinical and human clinical trials as a vaccine candidate against different viral, bacterial and parasitic infectious diseases.

**Table 6:** Preclinical and clinical trials using MVA vector as a vaccine candidate against different viral, bacterial and parasitic infectious diseases.

Target Pathogen or Disease	Heterologous Antigen	Status	References
**Viral diseases**			
Variola (smallpox)	Whole MVA vector	clinical	[299,300,311]
HIV/AIDS	HIV-1 Gag p24 and p17 fused to 25 overlapping CTL CD8 T cell epitopes (clade A)	clinical	[312]
	HIV-1 Env (clade E); Gag-Pol (clade A)	clinical	[313]
	HIV-1 Env, Gag, Tat-Rev and Nef-RT (clade C)	clinical	[314]
	HIV-1 Env, Gag-Pol, Nef-Tat (clades B/C)	clinical	[315]
	HIV-1 Gag, PR, RT, Env (clade B)	clinical	[316]
	HIV-1 Env/Gag, Tat/Rev/Nef-RT (clade B)	clinical	[127]
	HIV-1 Env, Gag-Pol-Nef (clade B)	clinical	[305]
	21 CTL and 18 HTL epitopes from HIV-1 Gag, Pol, Vpr, Nef, Rev and Env	clinical	[317]
	HIV-1 Nef	clinical	[318]
Influenza A	NP+M1	clinical	[319]
	HA from H5N1	clinical	[320]
Hepatitis B	HBs	clinical	[321]
	30 CTL and 16 HTL epitopes	preclinical	[322]
Hepatitis C	NS3, NS4 and NS5B (genotype 1b)	preclinical and clinical	[323,324]
	E1 and E2 (genotype 1b)	preclinical	[325]
	C, E1 and E2, p7, NS2, NS3, NS4A, NS4B, NS5A and NS5B (genotype 1a)	preclinical	[326]
Chikungunya	C, E3, E2, 6K and E1	preclinical	[327]
	E3 and E2	preclinical	[328]
	E3-E2, 6K-E1 or E3-E2-6K-E1	preclinical	[329]
Dengue	Envelope (Dengue type 2 virus)	preclinical	[330]
	Envelope (Dengue type 3 virus)	preclinical	[331]
Ebola	GP (Zaire and Sudan strains)	preclinical	[332]
CCHF	GP	preclinical	[333]
SARS	Spike protein	preclinical	[334,335,336,337]
	Spike or nucleocapsid proteins	preclinical	[338]
	Nucleocapsid	preclinical	[339]
MERS	Spike protein	preclinical	[340]
FIPV	Membrane protein	preclinical	[341]
RSV	F or G glycoprotein	preclinical	[342,343,344]
Rift valley fever	Gn/Gc GP	preclinical	[345,346]
Rabies	Glycoprotein	preclinical	[347]
JEV	Membrane (prM) and envelope (E) proteins (Korean strain)	preclinical	[348,349,350]
	B cell, CTL and Th multiple linear epitopes (SA14 strain)	preclinical	[351]
Measles	HA	preclinical	[352]
	F and HA	preclinical	[94,353]
CMV	Soluble GP B (gB)	preclinical	[354]
	UL55 (surface glycoprotein), UL83 (tegument protein) and UL123/e4 (nuclear protein)	preclinical	[355]
	pp65 (tegument protein) and CMV immediate early gene IE1	preclinical	[356]
	pp65-2, gB and IE1 (Rhesus CMV)	preclinical	[357,358]
	pp65, IE1, IE2	preclinical	[359]
	pp65	preclinical	[360]
	glycoproteins gH/gL, UL128, UL130 and UL131A (UL128C)	preclinical	[361]
	gH, gL, UL128, UL130 and UL131A	preclinical	[362]
BoHV-1	Secreted GP D	preclinical	[363]
EHV-1	Complement-receptor GP C	preclinical	[364]
HSV	GP D (gD) (HSV-2)	preclinical	[365]
Parainfluenza virus	F and/or HN glycoproteins (parainfluenza virus 3)	preclinical	[366,367,368]
**Bacterial diseases**			
Tuberculosis	Mycobacterial mycolyl-transferase antigen 85A	clinical	[369,370,371]
Babesia bovis	MSA-2c, RAP-1 and HSP20 proteins	preclinical	[372]
Bubonic plague	*Yersinia pestis* low-calcium response V antigen	preclinical	[373]
**Parasitic diseases**			
Malaria	ME-TRAP	clinical	[374,375]
	AMA1	clinical	[376,377,378,379,380]
	MSP1	clinical	[377,378,379,381]
	CS	clinical	[375,382]
	Polyprotein consisting of six pre-erythrocytic antigens from *P. falciparum*	clinical	[383]
Leishmaniasis	LACK	preclinical	[88,271,384,385,386]
	TRYP (*Leishmania major* substrain LV39)	preclinical	[387]
	TRYP (Leishmania *infantum)*	preclinical	[388]
	TRYP (*Leishmania (Viannia) panamensis*)	preclinical	[389]
Toxoplasmosis	ROP2	preclinical	[390]
Chagas disease	*Trypanosoma cruzi* TcG2 and TcG4	preclinical	[391]

Abbreviations: CTL: Cytotoxic T cell; HTL: Helper T lymphocyte; NP: Nucleoprotein; M1: Matrix protein 1; HA: Hemagglutinin; HBs: Hepatitis B surface antigen; GP: Glycoprotein; NHP: Non-human primates; CCHF: Crimean-Congo hemorrhagic fever; SARS: Severe acute respiratory syndrome; MERS: Middle East Respiratory Syndrome*;* FIPV: Feline infectious peritonitis virus; RSV: Respiratory syncytial virus; F: Fusion protein; JEV: Japanese encephalitis virus; CMV: Cytomegalovirus; BoHV-1: Bovine herpesvirus-1; EHV-1: Equine herpesvirus type 1; HSV: Herpes simplex virus; HN: Hemagglutinin-neuraminidase; ME-TRAP: Multiple epitope-thrombospondin-related adhesion protein; AMA1: *P. falciparum* blood-stage malaria antigen apical membrane antigen 1; MSP1: *P. falciparum* blood-stage malaria antigen merozoite surface protein 1; CS: Circumsporozoite protein; LACK: Leishmania homologue of receptors for activated C kinase; TRYP: Tryparedoxin peroxidase; ROP2: Toxoplasma gondii rhoptry protein 2.

### 5.5. Attenuated Avipoxviruses

Similar to VACV-derived strains, when considering the development of an APV-derived vector for production of a vaccine for birds, the use of an attenuated strain is recommended to reduce the risk and consequences of environmental spread to other avian species. Attenuated derivatives of FWPV (such as TROVAC or FP9) and CNPV (such as ALVAC) have been extensively tested, demonstrating their safety in a variety of species, including immunocompromised animals and human volunteers. Despite the fact that their multiplication is restricted to avian species, attenuated strains of APVs have been demonstrated to be efficacious and extremely safe vectors for mammals. Indeed, it was discovered that inoculation of APV-based recombinants into mammalian cells resulted in expression of the foreign gene and that inoculation into mammals resulted in the induction of protective immunity [103,172] (see Table 7).

**Table 7:** Vaccine applications of attenuated avipoxvirus-based vectors.

Poxvirus Strain	Target Pathogen or Disease	Heterologous Antigen	Status	References
**Viral infections**				
TROVAC	AIV	HA	licensed for commercial veterinary use (chickens)	[153,405,406,407,408]
	NDV	F and HN	preclinical	[409]
ALVAC	HIV/AIDS	SIV_K6W_ Gag-Pol-Env, Gag-Pol	preclinical	[118,410]
		HIV-1_IIIB_ Env	preclinical	[411]
		HIV-1_MN_ gp160	clinical	[412,413,414,415,416]
		HIV-1_MN_ gp120 linked to TM domain of HIV-1_LAI_ gp41, HIV-1_LAI_ Gag and protease	clinical	[417,418,419,420,421,422,423,424]
		HIV-1_MN_ gp120 linked to TM domain of HIV-1_IIIB_ gp41, HIV-1_IIIB_ Gag and protease, 3 CTL-dense regions of HIV-1_LAI_ pol, 2 CTL-dense regions of HIV-1_LAI_ nef	clinical	[425]
		CRF01_AE gp120 (92TH023) linked to TM domain of HIV-1_LAI_ gp41, HIV-1_LAI_ Gag and protease	clinical	[404,426,427,428,429,430,431,432]
	CMV	gB	clinical	[433,434]
		pp65	clinical	[435]
	Rabies virus	Glycoprotein	licensed for commercial veterinary use (cats)	[436]
			clinical	[437,438]
	CDV	HA and F	licensed for commercial veterinary use (dogs, ferrets)	[439,440,441,442]
	West-Nile virus	PrM-E	licensed for commercial veterinary use (horses)	[443,444,445,446,447]
	FeLV	Env, Gag	preclinical	[448]
		Env, Gag/pol	licensed for commercial veterinary use (cats)	[448–450]
	FIV	FIV _Ville Franche_ (subtype A) Env, Gag and Protease	preclinical	[451]
	EIV	HA	licensed for commercial veterinary use (horses)	[452,453,454]
	EHV	gB, gC and gD	preclinical	[455]
	JEV	prM, E, NS1	clinical	[456,457]
	HTLV-1	Env	preclinical	[458]
	AHSV	VP2 and VP5	preclinical	[459,460]
	RHDV	capsid protein	preclinical	[461]
	HCV	capsid, E1, E2, NS2, NS3	preclinical	[462]
	BTV	VP2 and VP5	preclinical	[463]
	AIV	HA	preclinical	[408,464]
**Bacterial diseases**				
FP9	Tuberculosis	Ag85A	preclinical	[465]
**Parasitic diseases**				
FP9	Malaria	CS	preclinical	[466]
		L3SEPTL (six-linked pre-erythrocytic antigens)	preclinical	[467]
		ME-TRAP, CS	clinical	[468,469,470,471,472,473,474]
ALVAC	Malaria	CS, SSP2, LSA1, MSP1, SERA, AMA1, Pfs25 CS, SSP2, AMA1, MSP1	preclinical	[475,476]

Abbreviations: AIV: Avian influenza virus; NDV: Newcastle disease virus; CMV: Cytomegalovirus; CDV: Canine distemper virus; FeLV: Feline leukemia virus; FIV: Feline immunodeficiency virus; EIV: Equine influenza virus; EHV: Equine herpes virus; JEV: Japanese encephalitis virus; HTLV-1: Human T cell leukemia/lymphoma virus type 1; AHSV: African horse sickness virus; RHDV: Rabbit hemorrhagic disease virus; HCV: Hepatitis C virus; BTV: Bluetongue virus; F: Fusion protein; HN: Hemagglutinin-neuraminidase proteins; HA: Hemagglutinin; TM: Transmembrane; gB: Glycoprotein B; pp65: Phosphoprotein 65; CS: Circumsporozoite protein; ME-TRAP: Multiple epitope-thrombospondin-related adhesion protein; SSP2: Sporozoite surface protein 2; LSA-1: Liver stage antigen 1; MSP1: Merozoite surface protein 1; SERA: Serine repeat antigen; AMA1: Apical merozoite antigen 1.

#### 5.5.1. TROVAC

For the generation of TROVAC, the attenuated fowlpox vaccine strain FP-1 [103], derived from the Duvette strain, was subjected to four successive plaque purifications. Then, one plaque isolate was further amplified in primary CEF cells and a viral stock, designated as TROVAC, was generated and deposited with the American Type Culture Collection (ATCC; Accession number: VR-2553) [392].

#### 5.5.2. FP9

FP9 is a highly attenuated form of FWPV derived from the wild-type fowlpox virus HP-1 by 438 serial passages in CEF cells [393]. The FP9 genome has been fully sequenced and found to harbor several deletions/insertions and gene modifications when compared with the sequence of wild-type FWPV strains [394].

#### 5.5.3. ALVAC

ALVAC is a plaque-purified clone derived from an attenuated CNPV obtained from the wild-type strain after 200 serial passages in CEF cells [395]. It has been extensively evaluated in preclinical studies with non-human primates [396,397,398,399,400], widely used in human clinical trials as an HIV/AIDS vaccine candidate [109,401], and licensed for veterinary use [111]. ALVAC-based vectors have been reported to be well tolerated and safe for humans [402,403] and the first sign of efficacy of an HIV/AIDS vaccine candidate, although modest (31.2%), was obtained in a phase III clinical trial using an ALVAC-based vector [404].

## 6. Fourth-Generation VACV Vaccines: Evolution through Genetic Engineering

The innovative biotechnology techniques of genetic engineering that have been developed in the 1980s and 90s allow the generation of novel poxvirus vaccines through the insertion or deletion of specific genes in the poxvirus genome. In 1982, two independent groups showed for the first time that VACV can be modified to be used as an expression vector system, where foreign DNA can be inserted into non-essential regions of the VACV genome [13,14]. Since then several recombinant poxviruses have been generated and used as effective vector systems for vaccination-expressing heterologous antigens that were able to induce strong antigen-specific cellular and humoral responses, reinforcing the use of recombinant poxviruses as vaccine candidates against a broad range of infectious diseases. Numerous replication-deficient and competent poxvirus-based vectors have been widely and successfully used as vaccine candidates in preclinical and clinical trials in the prevention and treatment of different animal and human diseases (reviews in [37,301,302,303,304,305,306,307,308,309,310,477,478,479,480]). The genetic modifications normally attenuated the virus and led to an increase in immunogenicity against the VACV antigens or against the heterologous antigens expressed from the poxvirus vector.

### 6.1. Deletion of Genes

Poxviruses encode for many proteins involved in the host innate immune evasion, with secreted proteins that bind and neutralize IFNs, cytokines and chemokines, or intracellular proteins that inhibit apoptosis or signaling pathways that lead to the production of IFNs or proinflammatory cytokines and chemokines [289]. Thus, deletion of these VACV genes involved in immune-modulation, host-range and accessory nucleotide metabolism genes is one of the techniques that has been widely used to generate novel poxvirus vectors with a more attenuated profile or novel vaccine candidates with optimized immunogenicity [481].

One of the best examples of an attenuated VACV vector generated by the deletion of viral genes is NYVAC, a VACV strain derived from a plaque-cloned isolate (VC-2) of the Copenhagen strain (VACV-COP) by the precise deletion of 18 Open Reading Frames (ORFs). These deleted genes include 12 ORFs from *C7L* to *K1L* genes, *J2R (TK)*, *B13R*, *B14R*, *A26L*, *A56R (HA)* and *I4L* and are involved in pathogenicity, virulence and host-range functions [482]. The resultant vector exhibits a dramatically reduced ability to replicate on a variety of human and mammalian cell types, is highly attenuated since it fails to disseminate in immunodeficient mice, and is unable to produce infectious virus in humans [482,483,484]. Despite its limited replication in human and most mammalian cells, NYVAC provides a high level of gene expression and triggers antigen-specific immune responses when delivering foreign proteins in animals and humans [485,486,487,488]. For this reason, NYVAC-based recombinants are underintensepreclinical and clinical investigation as recombinant vaccines against multiple infectious diseases [305,306,483] (see Table 8).

**Table 8:** Vaccine applications of fourth-generation poxvirus-based vectors generated by the deletion of poxviral genes.

Poxvirus Strain	Target Pathogen or Disease	Heterologous Antigen	Status	References
**Viral infections**				
NYVAC	PRV	Glycoprotein, gB or gD glycoproteins, gII, gIII and/or gp50 glycoproteins	preclinical	[483,489,490,491,492,493,494]
	CDV	F and HA	preclinical	[440,441,442]
	EHV	gene 64	preclinical	[455]
	JEV	prM, E, NS1	clinical	[456,457]
	HIV/AIDS	SIV_K6W_ Env-Gag-Pol, SHIV_89.6P_ Env, SIV_mac239_ Gag-Pol-Nef	preclinical	[487,495,496,497]
		Env (clade B)	preclinical	[411]
		clade C trimeric soluble gp140(ZM96), clade C Gag(ZM96)-Pol-Nef(CN54) as VLPs	preclinical	[498]
		Env, Gag-Pol-Nef (clade C)	clinical	[499,500,501,502,503]
		Env, Gag-Pol-Nef (clade B)	clinical	[504,505]
	AIV	HA	preclinical	[408]
	HTLV-1	Env, Env + Gag	preclinical	[458,506,507]
**Parasitic diseases**				
NYVAC	Malaria	LSA-1, CS	preclinical	[508]
		CS, SSP2, LSA1, MSP1, SERA, AMA1, Pfs25	clinical	[509]

Abbreviations: PRV: Pseudorabies virus; AIV: Avian influenza virus; CDV: Canine distemper virus; EHV: Equine herpes virus; JEV: Japanese encephalitis virus; HTLV-1: Human T cell leukemia/lymphoma virus type 1; F: Fusion protein; HA: Hemagglutinin; CS: Circumsporozoite protein; SSP2: Sporozoite surface protein 2; LSA-1: Liver stage antigen 1; MSP1: Merozoite surface protein 1; SERA: Serine repeat antigen; AMA1: Apical merozoite antigen 1.

Most of the studies involved in the deletion of immune-modulating VACV genes have been performed in the VACV WR strain and the general results showed that deletion of many VACV genes attenuated the virus [289], but the impact on immunogenicity was variable. Thus, deletion of some immunomodulatory VACV genes from different strains (mainly WR and MVA) increased the immunogenicity against VACV antigens, as it is described for VACV genes *E3L* [510], *B15R/B16R* [511,512,513], *A41L* [514], *B13R* and *B22R* [515], *C12L* [516], *A35R* [517] or *C6L* [518]. However, deletion of other immunomodulatory genes has no effect on the virulence or pathogenicity, as in *B8R* [519,520], or does not enhance the immunogenicity against VACV, as in *N2L* [521] or *C16L* [522]. Moreover, deletions of *C12L*, *A44L*, *A46R* or *B7R* in MVA did not significantly affect VACV-specific CD8 T cell immunogenicity in BALB/c mice [511]. Furthermore, the Wyeth strain with deletions of coding regions for the *B5R*, *B8R*, *B12R*, *B13R*, *B14R*, *B16R*, *B18R* and *B19R* immunomodulatory gene products did not increase the immunogenicity of these vectors compared with the parental VACV [523].

Recently, several deletions in the Tian Tan strain have been performed and analyzed. For example, a recombinant Tian Tan VACV expressing HIV-1 Gag, Pol and Env genes and with deletions in the *C12L* and *A53R* genes is highly attenuated and retains the high immunogenicity of the parental virus to elicit strong humoral and cellular responses to the HIV-1 target genes [524].

In addition, it has been reported that deletion in the Lister strain of the five major nonessential regions that are deleted in MVA enhances the attenuation, although the VACV-specific immune responses were similar to those of the traditional smallpox vaccine [525]. Nonetheless, introduction of the six major genomic deletions of MVA into the parental VACV CVA is not sufficient to reproduce an MVA-like phenotype in cell culture and in mice [526].

Combination of the insertion of a heterologous antigen in a poxvirus vector with the deletion of an immunomodulatory VACV gene is a promising novel approach to optimize the poxvirus vaccine vector by enhancing immunogenicity against the foreign antigen [481]. This strategy has been widely used for the generation of optimized recombinant MVA and NYVAC vectors expressing HIV-1 antigens (which are used as HIV/AIDS vaccine candidates) and containing single or multiple deletions in immunomodulatory VACV genes that antagonize host-specific immune responses. These new optimized recombinant MVA and NYVAC vaccine vectors lacking VACV immunomodulatory genes have been tested in mice [327,516,527,528,529,530,531,532] and non-human primates [533,534], and the overall results showed that they induced an enhancement in the HIV-1-specific cellular and humoral immune responses.

Thus, the removal of VACV immunomodulatory genes that block the host immune responses to the infection is a useful method to enhance the antigen-specific immune responses induced by different poxvirus-based vaccine candidates. Dissection of the immune profile induced by these novel poxvirus vectors with deletions in single genes or in gene families is necessary to find an optimized poxvirus vector that could enter into future human clinical trials to test whether it can provide protection against infection. In fact, system biology profiles of NYVAC vectors expressing HIV-1 antigens and lacking the IFN inhibitors B8 and B19 revealed in human macrophages distinct gene signatures that can be correlated with immune parameters relevant in protection [535]. Gene signatures have also been defined for the HIV vaccine candidates MVA-B [291] and MVA-C [536]. A summary of poxvirus genes deleted in the context of poxvirus-based vaccine vectors against different infectious diseases is shown in Table 9.

**Table 9:** Poxvirus genes deleted in poxvirus-based vaccine candidates against infectious diseases that enhance the vector immunogenicity.

Pox Strain	Target Pathogen or Disease	Heterologous Antigen	Poxvirus Deleted Gene	Gene Function	Status	References
MVA	HIV/AIDS	HIV-1 Env, Gag-Pol-Nef (clade B)	*A41L/B16R*	*A41L*: Secreted chemokine-binding protein *B16R*: Secreted interleukin-1β binding protein	preclinical	[528]
		HIV-1 Env, Gag-Pol-Nef (clade B)	*C6L*	IRF3 inhibitor	preclinical	[527,529]
		HIV-1 Env, Gag-Pol-Nef (clade B)	*C6L/K7R*	*C6L*: IRF3 inhibitor *K7R*: NF-кB/IRF3 inhibitor	preclinical	[527]
		HIV-1 Env, Gag-Pol-Nef (clade B)	*N2L*	IRF3 inhibitor	preclinical	[537]
		HIV-1 Env, Gag-Pol-Nef (clade C)	*F1L*	Anti-apoptotic protein	preclinical	[532]
		HIV-1 Env, Gag-Pol-Nef (clade C)	*C12L*	IL-18 binding protein	preclinical	[516]
		HIV-1 Env, Gag (clade C)	*C12L/A46R/B7R/B16R*	*C12L*: IL-18 binding protein *A46R*: Inhibitor of TLR signaling *B7R*: Putative chemokine-binding protein *B16R*: Secreted interleukin-1β binding protein	preclinical	[533]
NYVAC	HIV/AIDS	HIV-1 Env, Gag-Pol-Nef (clade C)	*B8R*	Secreted IFNγ binding protein	preclinical	[530]
		HIV-1 Env, Gag-Pol-Nef (clade C)	*B19R*	Type I IFN binding protein	preclinical	[530]
		HIV-1 Env, Gag-Pol-Nef (clade C)	*A46R*	Inhibitor of TLR signaling	preclinical	[531]
MVA	Chikungunya	C-E1-E2-6K-E3	*C6L/K7R/A46R*	*C6L*: IRF3 inhibitor *K7R*: NF-кB/IRF3 inhibitor *A46R*: Inhibitor of TLR signaling	preclinical	[327]
MVA	Smallpox	-	*A35R*	Inhibitor of MHC class II antigen presentation	preclinical	[517]
MVA	Smallpox	-	*A41L*	Secreted chemokine-binding protein.	preclinical	[514]
MVA	Smallpox	-	*C6L*	IRF3 inhibitor	preclinical	[518]
MVA	Smallpox	-	*B16R*	Interleukin-1β binding protein	preclinical	[511,512,513]
NYCBH	Smallpox	-	*E3L*	dsRNA-binding protein		[538,539,540]
FWPV	AIV	HA	ORF73 or ORF214	Suggested interleukin-18 binding proteins	preclinical	[541]

Abbreviations: AIV: Avian influenza virus; HA: Hemagglutinin.

### 6.2. Insertion of Genes

One of the advantages of replication-deficient viruses is their safety profile. However, it has been postulated that the efficacy of these viruses is restricted due to the failure to replicate and the limitation in antigen accumulation during virus infection. For this reason, the restoration of replication competence in human cells, together or not with the deletion of specific immunomodulatory VACV genes, can be a strategy to improve the efficacy of poxvirus-based vectors.

In the case of the NYVAC vector, the restoration of replication capacity is obtained by the reinsertion of *K1L* and/or *C7L* host range genes in the viral genome. It has been reported that these new constructs are still attenuated but acquire new biological properties distinct from the parental NYVAC that make them potentially improved vaccine vector candidates for human applications [542,543] (see Table 10). Furthermore, the gene signatures of a replication-competent NYVAC vector expressing HIV-1 genes (termed NYVAC-C-KC) in human dendritic cells have been described [542].

Another category of genes that has been used to improve poxviruses as vaccine vectors is those that encode co-stimulatory molecules such as IL-1α, IL-2, IFN-γ, IL-12, IL-15, OX40/OX40L, B7-1, ICAM-1, LFA-3, CD80, CD86, CD83, CD40L or GM-CSF [481]. This strategy significantly enhanced the immunogenicity and efficacy of the poxvirus vector as a vaccine against different infectious diseases and has been extensively used against cancer (see Section 7). However, there is a limitation on the insertion of this immunomodulators. Ramshaw and colleagues discovered in 2001 that the insertion of mouse interleukin-4 by a recombinant ectromelia virus suppressed antiviral cell-mediated immune responses [544]. This has been further explored and the insertion of this Th2 cytokine into several poxviruses significantly increased the efficiency of the recombinant virus as a pathogen by directly inhibiting the development of Th1 immunity, which is crucial for viral clearance [545,546].

**Table 10:** Vaccine applications of fourth-generation poxvirus-based vectors generated by the insertion of viral genes.

Poxvirus Strain	Target Pathogen or Disease	Heterologous antigen	Inserted Gene	Gene Function	Status	References
**Viral infections**						
NYVAC	HIV/AIDS	HIV-1 Env, Gag-Pol-Nef (clade C)	VACV *K1L* and *C7L* (*B19R* deletion)	Host range	preclinical	[542,543]
		HIV-1 Env, Gag-Pol-Nef (clade B)	VACV *C7L*	Host range	preclinical	[547]
	MeV	HA	VACV *K1L*	Host range	preclinical	[548]
ALVAC	HIV/AIDS	HIV-1_MN_ gp120 linked to TM domain of HIV-1_LAI_ gp41, HIV-1_LAI_ Gag and protease, synthetic polypeptide encompassing several human *nef* and *pol* epitopes, CD40L	VACV *E3L* and *K3L*	PKR and/or 2'5'OAS inhibitors	clinical	[549,550,551,552,553,554,555,556]
**Parasitic diseases**						
NYVAC	Malaria	CS	VACV *K1L*	Host range	preclinical	[557]

Abbreviations: MeV: Measles virus; HA: Hemagglutinin; TM: Transmembrane; PKR: Double-stranded RNA-dependent protein kinase; 2'5'OAS: 2'-5' oligoadenylate synthetase; CS: Circumsporozoite protein.

### 6.3. Gene Expression Optimization

The optimization of gene expression of poxvirus-based vaccines is addressed to improve the generation of immune responses to the heterologous antigen. Thus, the regulation of the antigen expression level is an alternative vaccine-design strategy adopted to induce antigen-specific immune responses [558].

In this regard, the late-early VACV p7.5 promoter [559] was the first strategy used to induce heterologous antigen expression. The removal of poxvirus transcription termination signals from inserted genes [560] and the regulation of gene expression under the bacteriophage T7 promoter [561], the vaccinia modified H5 (mH5) [366] and the vaccinia short synthetic early-late pS [562] promoters have been used as alternatives to p7.5 to increase the quantity of heterologous antigen expressed during infection.

Recently, it has been demonstrated that the efficiency with which an antigen is processed and presented on the surface of infected cells influences its recognition [563]. In fact, in VACV, 90% of the antigens most recognized by CD8 T cells were ranked among the top 50% in terms of mRNA expression [564], and there is a correlation between the timing of viral antigen expression and the generation of antigen-specific immune responses [565]. For this reason, efforts towards developing new poxvirus vaccines candidates are focused on using promoters to improve the timing rather than the quantity of antigen expression.

After a deep analysis of the VACV transcriptome, two groups have defined two categories of early genes based on their temporal expression [566,567]. Based on these studies, endogenous poxviral early promoters have been compared with the p7.5 and pS promoters. The pC11R and pF11L promoters induced high levels of early antigen expression and cellular immunogenicity similar to those of p7.5 and pS promoters [568].

More recent studies demonstrate that it is possible to design poxvirus promoters that improve early antigen expression and antigen-specific T cell responses. In this regard, synthetic early promoters such as psFJ1-10 [569,570] or pHyb [571] and native early promoters like PrMVA13.5-long [572] present repeated motifs, each containing an early promoter element. An alternative strategy for poxvirus promoter design is the optimization of the early promoter element after bioinformatic analysis, as indicated by the Late-Early Optimized (LEO) promoter [573]. These new promoters are able to increase the expression of heterologous antigens and their specific immune response compared to the p7.5 and pS. They represent excellent prototypes for the generation of safe poxvirus recombinant-based vaccines to potentiate the antigen expression and immune response.

## 7. Poxvirus Evolution as Vaccines to Fight Cancer

Poxviruses represent strong contenders for cancer vaccine development given their ability to express large foreign genes, capacity to induce a strong cytotoxic T lymphocyte (CTL) responses, broad tissue tropism, fast replication and lysis of infected cells, potential to take advantage of the tumor microenvironment (deregulation of cell cycle control, partially blocked IFN response and apoptosis or immune evasion), and the absence of DNA integration into the host genome for safety [574].

All these features could be a potential solution to a range of issues that characterize cancer: low immune response generated by tumor-associated antigens (TAA), strong immune-suppressor tumor environment, antiviral immune response elicited by the vector and concerns regarding the safety of the vaccine used (sites of virus infection and/or replication, toxicity of the transgene expressed, or other vaccine-associated side effects) [575,576].

In 1963, for the first time, a poxvirus was assayed as a potential vector to treat tumors; in this case, Purified Vaccine Lymph was used to treat various skin cancers by local injection [577]. Within the several poxvirus-based strategies deployed and analyzed at preclinical stages for cancer vaccine development, VACV, FWPV, CNPV and their combinations as vectors represent the majority seen on Table 11.

As we have described for infectious diseases, in the development of different vaccines against cancer, various approaches involve the insertion of heterologous genes into common poxvirus strains, such as immunotherapy by the expression of TAA (e.g., MUC1 [578], oncofetal antigen 5T4 [579], PSA [580], CEA [581]), the expression of immunomodulatory genes (e.g., costimulatory molecules-B7.1, B7.2 [582], CD80 [583] or cytokines-IL-2 [584], IFN-β [585], GM-CSF [586]), the expression of suicide genes (e.g., cytosine deaminase [587], purine nucleoside phosphorylase [588]), and the expression of genes used for the imaging as a support for combination therapies (thyroidal sodium iodide symporter NIS [589]).

On the other hand, a different artificial evolution of poxviruses has been performed to direct the natural oncolytic capacity of this family, generating tumor-tailored viruses that grow to a higher extent in tumor cells and microenvironment. In this way, poxviruses have been engineered by the deletion of specific genes involved in nucleotide metabolism, interferon response, the cell cycle and other cell functions abnormally regulated in tumor cells (e.g., *J2R*, *C11R*, *B18R* [574]).

Likewise, combinations of all the different strategies mentioned above have been evaluated altogether in different immunization protocols. Some of these strategies are especially promising, which is reflected in the significant number of human clinical trials in phase I, II or III that have been or are being carried out targeting different types of cancers such as melanoma, breast, prostate or liver cancer [479].

**Table 11:** Poxvirus-based vaccine candidates against cancer.

Poxvirus	Strategy	Strain	Gene	Status	References
Vaccinia virus	TAA	MVA, Copenhagen	Deletion: *J2R*, *A56R*, *IGR3*	preclinical	[578,579,580,590,591,592,593,594,595,596,597,598,599,600]
			Neu oncogene; MUC1; oncofetal antigen 5T4; tumor-associated auto-antigen p53; PSA; PSCA; STEAP1; GA733 Ag; AFP; murine surviving; HPV-16 E1 oncoprotein; EBNA1-LMP2		
	Immunomodulation	NYCBH, MVA, Wyeth	Deletion: *J2R*	preclinical	[479,586,601,602,603,604,605]
			GM-CSF; IL-2		
	TAA + Immunomodulation	MVA, WR, Copenhagen	Deletion: *J2R*, *A56R*, *I4L*, *A44L*	preclinical	[479,582,583,584,606,607]
			TAA: MUC1, Melan-A/Mart-_1 27-15_ minigene; gp100_280–288_ + Melan-A/MART-_127–35_ + tyrosinase_1–9_ tumor-associated antigen epitopes; HER-2		
			IL-2; costimulatory molecules B7.1 and B7.2; CD80 and CD86; p2 and p30 T helper cell epitopes from tetanus toxin		
	Oncolysis	WR, Copenhagen, Wyeth, MVA, Lister, LIVP	Deletion: *J2R*, *C11R*, *B18R*, *F14.5L* Mutation: *A34L*, *A5L*	preclinical	[581,587,588,589,608,609,610,611,612,613,614,615]
			hNIS; CEA; Neu oncogene; ETA; polyomavirus-specific tumor-specific antigens; early bovine papillomavirus proteins; CD; PNP; FCU1; RUC-GFP; TFR		
	Oncolysis + Immunomodulation	WR, Wyeth	Genes: *J2R, C11R, B18R, A56R*	preclinical	[479,585,616,617,618,619,620,621,622,623,624,625,626]
			VEGF-binding ectodomain from Flk1; T-cell engager EphA2-TEA; GM-CSF; IFN-β; CCL5 (RANTES); IL-2; IL-12 (p35 and p40 subunits); FasL; CXCR4; CD40L		
Fowlpox virus	TAA	FWPV	HPV-16 E6 and E7 oncoproteins	preclinical	[479,627]
	TAA + Immunomodulation	FWPV	TAA: PAP	preclinical	[628]
			IL-2		
Canarypox	TAA	ALVAC	tumor-associated auto-antigen p53; gp100, MAGE-1,3 minigene; NY-ESO-1; MART-1	preclinical	[479,629,630]
	TAA + Immunomodulation	ALVAC	TAA: CEA; gp100 protein	preclinical	[479,631,632,633,634,635]
			costimulatory molecule B7.1		
	Immunomodulation	Myxoma	Fusion Protein of Interleukin-15 (IL15) and IL15 Receptor Alpha	preclinical	[636]
	Oncolysis	Myxoma	Gene insertion: VACV *F11L*	preclinical	[637,638]
Mix	TAA	VACV/FWPV	NY-ESO-1; Tyrosinase	preclinical	[639,640]
	Immunomodulation	VACV (NYVAC)/CNPV (ALVAC) FWPV/Canarypox (ALVAC)	IL-2; GM-CSF	preclinical	[479,641,642]
	TAA + Immunomodulation	VACV/FWPV; VACV (Wyeth)/CNPV (ALVAC)	TAA: PSA; CEA; MUC1	preclinical	[479,643,644,645,646,647,648,649]
			TRICOM; GM-CSF; IL-2		
Parapox virus	Oncolysis	NZ2	vascular endothelial growth factor locus	preclinical	[650]
Raccoonpox virus	Oncolysis	Not described	GFP (TK locus)	preclinical	[651]
YLDV (yatapoxvirus)	Oncolysis	Not described	GFP (TK locus)	preclinical	[652]

Abbreviations: IGR3: Alternative insertion site known as intergenic region 3—located in the Hind III I region (between *I3L* and *I4L*); MUC1: Mucin-1; PSA: Prostate-specific antigen; PSCA: Murine prostate stem cell antigen; STEAP1: Murine six transmembrane epithelial antigen of the prostate 1; GA733 Ag: Glycoprotein GA733/CO17-1A/EpCAM/KSA/KS1-4; AFP: A-fetoprotein; HPV: *Human papillomavirus*; EBNA1-LMP2: CD4 epitope-rich C-terminal domain of EBNA1 fused to full-length LMP2; GM-CSF: Granulocyte–macrophage colony-stimulating factor; IL-2: Interleukin 2; HER-2: Human epidermal growth factor receptor 2; hNIS: Human thyroidal sodium iodide symporter; ETA: Epithelial tumor antigen; CD: Cytosine deaminase; PNP: Purine nucleoside phosphorylase; FCU-1: Fusion yeast CDaseH-UPRTase gene; TFR: Human transferrin receptor; IFN-β: Interferon-β; CD40L: CD40 ligand; PAP: Prostate tumor-associated antigen prostatic acid phosphatase; CEA: Carcinoembryonic antigen; TRICOM: Costimulatory molecules (B7.1, ICAM-1 and LFA-3); GFP: Green fluorescent protein; YLDV: Yaba-like disease virus.

## 8. Future Perspectives

Since Jenner first described in 1798 the application of a virus isolated from a cow to demonstrate the efficacy of vaccination against smallpox, the poxvirus family has been in constant change and under human-made adaptation. It was soon realized that this family of viruses was quite large and infected a wide range of animal species. Only the orthopoxvirus genus replicates productively in humans, with the variola strain being the cause of smallpox. Due to the health problems inherent in smallpox, one of the most dreadful diseases of human mankind with a death rate of about 30%, a major effort was dedicated to eradicate this scourge. It was not until 1980 that the WHO declared that the world was free of smallpox. Along the way, many studies aimed to understand the biology of this group of animal viruses, and major scientific discoveries emerged that had a profound effect on biology as a whole. In fact, scientific concepts as relevant as the basis of immunology and antibody responses to virus infection, the components of a virus particle (DNA, protein, lipids), the presence of a DNA-dependent RNA polymerase, the virion machinery for mRNA synthesis and modifications at the 3'-end and 5'-end, the formation of two forms of infectious virus particles, the existence of multiple viral genes with the capacity to counteract host immune responses, and the ability of the virus to accept the insertion of heterologous foreign genes in the viral genome or the removal of multiple viral genes, are among features that define the plasticity of this family of viruses, which in turn increased our knowledge of living viruses and cells.

The urgency to develop attenuated vaccines promoted the use of different animal models and cell culture systems for virus isolation. As a result a number of vaccine strains emerged in different countries. It was not until whole genome sequencing was developed that we realized the occurrence of different genetic changes within the virus genome. Being a large DNA with a high fidelity polymerase, it was not surprising that, in order to observe genetic alterations such as deletions and point mutations, multiple passages of the virus in cell cultures were needed for attenuation. This effort resulted in the isolation and identification of the now most widely used poxvirus vaccine strain candidates, mainly those derived from NYCBH (Dryvax and ACAM2000), Lister, LC16m8, Tian Tan, MVA, NYVAC and ALVAC.

After the first entire DNA sequence of the Copenhagen strain of VACV was described in 1990, the number of poxvirus whole genome sequences has increased considerably. The information provided facilitated the identification of mutations that correlated with an attenuated phenotype. Indeed, it was found that in the case of the LC16m8 strain derived from Lister, the cause of attenuation is a frame-shifting single nucleotide deletion in the *B5R* gene. Thus, the old approach of allowing the appearance of spontaneous mutations in the virus genome during cell passages is no longer the method of choice. In fact, as soon as knowledge developed on the biological role of viral genes, newly designed vectors were developed, like NYVAC, a vector derived from the Copenhagen strain by selective deletion of 18 open reading frames (ORFs). The DNA sequencing methods together with the easy method for removing or incorporating selected genes in the viral genome have considerably expanded our understanding of the role of viral-encoded immune modulators and the use of poxvirus vectors as vaccine candidates.

Multiple vaccine candidates have been developed based on members of the poxvirus family with the ability to express the genes of interest in hosts of different origins. The most widely used poxvirus vectors are derived from MVA, NYVAC and ALVAC, and while none of these vectors has been approved for human usage as a virus recombinant vaccine, the promising results obtained in a large number of preclinical and clinical trials presage a not-too-distant application of these recombinant viral vectors as vaccines in humans against multiple diseases. This is exemplified in the partial efficacy of ALVAC against HIV in a phase III clinical trial in Thailand, and in the recent outbreak of Ebola after efficacy results were observed in non-human primates with the prime/boost combination of adenovirus and MVA vectors expressing Ebola GP protein, a protocol that might be implemented as part of the phase I/II clinical trials that have been initiated at various sites in Africa. There is also abundant preclinical information on the proven efficacy of these vectors in other model diseases.

What can we expect the next steps in poxvirus vaccine evolution to be? The fact that the whole virus genome sequence can be reconstituted through synthesis of nucleotides, that the virus genome can be easily manipulated genetically, and that new information on the role of viral genes and interactions with the host cell are known, indicate that for vaccine purposes novel vectors with high specificity to trigger B and T cell immune responses and with high protective capacity will be developed. Indeed, novel vectors triggering high immune responses against the foreign expressed antigens have been generated, either by selective deletion of viral immune modulators, incorporation of host range genes, incorporation of cytokines/chemokines genes or of other inducers/activators of immune responses. Still it is unclear if we just need to develop vectors that trigger very potent immune responses, *i.e.*, high ELISPOT numbers, or just to select those that trigger long-term memory B and T cell responses as an index of potency. In all cases, efficacy will be needed in model systems, as well as definition of immune correlates of protection. The implementation of system biology approaches in preclinical and clinical trials, from non-human primates to vaccinated individuals, will identify gene signatures relevant in protection and could aid in the selection of optimal immunogens. Still, much remains to be learned about the biological role played by many of the virus-encoded immune modulators. For vaccine purposes, it will be important to advance with vectors that are well characterized in terms of pathogenicity, immunogenicity and molecular signatures. Best-in-class candidates can be defined by direct head-to-head comparison on immune characteristics among vectors. Since the final aim is to develop a vaccine that fully protects against a disease, this will only be known with the progressive advance of generating optimized poxvirus vectors and studying their behavior in animal models and in clinical trials. Nonetheless, it is predicted that while in some cases a single poxvirus recombinant vector might be sufficient to fight a disease, it is likely that in most cases heterologous vector combinations, like DNA, RNA, protein or other attenuated viral vectors, will be used together in prime/boost protocols to fight more complex diseases. Overall, we have seen remarkable changes in poxvirus evolution with time, from virus isolation in animals and cell cultures to selectively targeting viral genes. As more scientific information is gained on vector biology and preclinical and clinical trials further advance, showing health benefits on vectors’ behavior, we anticipate a bright future for the poxvirus-based vaccine field.
